# Intraoperative brain relaxation as a therapeutic target and proposal of a new definition

**DOI:** 10.1016/j.bas.2026.105939

**Published:** 2026-01-11

**Authors:** Ved Prakash Maurya, Luis Rafael Moscote-Salazar, Pratiksha Baliga, Moshiur Rahman, Tariq Janjua, Mariana Beltran Lopez, Amit Agrawal

**Affiliations:** a Department of Neurosurgery, Sanjay Gandhi Postgraduate Institute of Medical Science, Lucknow, India, 226014; b AV Healthcare Innovators, LLC, Madison, WI, USA; c Mahatma Gandhi Mission Institute of Health Sciences, India; d Neurosurgery Department, Holy Family Red Crescent Medical College, Dhaka, Bangladesh; e The National Brain Aneurysm & Tumor Center, Minneapolis, USA; f Department of Medicine, Universidad del Tolima, Ibague, Colombia; g Department of Neurosurgery, All India Institute of Medical Sciences Bhopal, Saket Nagar, Bhopal, 462020, Madhya Pradesh, India

**Keywords:** Dexamethasone, Intracranial pressure, Brain relaxation, Mannitol, Physiological

Dear Editor,

In 1962, Hayes and Slocum first addressed the issue of a swollen brain. They introduced the term ‘brain relaxation’ in the title of their paper. Other common terms such as cerebral relaxation, brain swelling, and brain herniation, which were proposed to describe such conditions (Hayes and Slocum, 1962; Jørgensen et al., 1999; Cold et al., 1996), have also been used. During brain surgeries, cerebral relaxation becomes an important aspect that determines the degree of retraction injury and influences the operative feasibility for surgeons. These factors can directly impact the patient's outcome (Randell and Niskanen, 2006; Andrews and Bringas, 1993). Despite its practical relevance, the standard definition, assessment, and management of intraoperative brain relaxation have not been thoroughly scrutinised. Most of the time, the assessment of brain bulge is subjective and mainly guided by the operating surgeon or the surgical team. This results in significant subjective or interobserver variability. Traditionally, osmotic therapy, such as mannitol and hypertonic saline, is the neurosurgical treatment for elevated intracranial pressure (ICP) (Boone et al., 2015; Prabhakar et al., 2014). They are used more promptly in cases of traumatic brain injury with features of raised intracranial pressure; >20 mmHg for more than 5 minutes.

The use of dexamethasone in the perioperative period to alleviate vasogenic oedema has been found to be notably beneficial in managing intraoperative brain bulge. Variations in the dosage of osmotic therapy (20 % Mannitol) complicate the situation further. The Brain Trauma Foundation recommends a dosage of 1.0 g/kg of mannitol. Conversely, in a country like Spain, the approved dose of 20 % mannitol ranges from 0.25 to 0.5 g/kg (Li et al., 2016; Fang et al., 2018). In clinical practice, these anti-oedema measures lack clear guidelines on the timing and dosage of administration. Most often, these agents are administered not after a measurable increase in ICP but after visual assessment by the surgeon following dural opening. This represents an evidence-practice gap for such a crucial case. We suggest that brain relaxation, in response to measurable cortical tension, protrusion, or swelling during durotomy, will become an unavoidable treatment target. By making decisions based on systematic and objective assessments of brain relaxation, rather than indirect inference or traditional practice, osmotherapy would be administered more logically and reproducibly (Li et al., 2016).

## 1 The concept of brain relaxation

Brain relaxation is the visual and palpable state of the brain after durotomy that indicates cortical swelling, intracranial compliance, and ICP dynamics. Several grading scales have been used, and among these, the modified 4-point scale of brain relaxation is one of them.

• Completely relaxed – cortex below skull level
• Satisfactorily relaxed – cortex at skull level
• Firm brain – slight bulging above skull
• Bulging brain – tense or herniating brain

The term brain relaxation describes a clinical state that involves several key factors, such as physiological and anaesthetic aspects, and it influences the outcome (Todd et al., 1993). Although this measurement is subjective, it is valuable in predicting operative complexity and may be more closely related to outcomes than isolated ICP values. The brain bulge should be viewed as the result of persistent or ongoing intracranial pressure. The bulging brain serves as an indirect sign of a potentially serious situation. If not addressed promptly, this condition may lead to worse outcomes.

## 2 Potentially modifiable factors in addressing brain bulge

### 2.1 Surgical position

An ergonomically appropriate position forms the foundation of an effective surgical procedure, which in turn ensures a smooth intraoperative phase. In cranial surgeries, excessive head rotation can cause venous congestion, leading to intraoperative brain swelling. In spinal surgeries, compressing the abdomen increases pressure in the splanchnic circulation, resulting in diffuse venous bleeding (Pérez de et al., 2022).

### 2.2 Intraoperative ventilation

To maintain adequate perfusion without causing hyperemia is the goal of optimal neuroanaesthesia. The ideal PEEP value should be kept at 4–5, while any anticipated brain bulge needs to be managed with prophylactic hyperventilation. The effects of hyperventilation are quickest to manifest and subside rapidly (Bruder et al., 2017).

### 2.3 Cerebrospinal fluid release

CSF, being a non-compressible substance, needs to be released quickly either via external ventricular drainage or through the natural cisternal spaces of the brain. It provides an instant and highly consistent solution to the brain bulge during the intraoperative period.

### 2.4 Pharmacological agents

The inhalation agents of choice should be selected carefully, and the decision to administer osmotic agents to address intraoperative brain bulge should be made well in advance. If used judiciously, these agents prevent cerebral ischemia by reducing the risk of the steel phenomenon. Mannitol has been used as a hyperosmolar therapy since 1960, but recent studies have emphasized that hypertonic saline is also equally effective in decreasing raised intracranial pressure.

## 3 Proposing a new approach

We propose a new definition of brain relaxation as the dynamic intraoperative condition. This state is a reflection of the ratio between the amount of intracranial content and the capacity of the cranial cavity following a craniotomy. Of course, it would be evaluated using visual grading and could even be a target for treatment. Another definition with great importance is that of brain relaxation, where a balanced ratio between the intracranial content and the capacity of the intracranial cavity is achieved. Brain relaxation is a concept that defines a perfect ratio between intracranial content and intracranial cavity capacity during intracranial surgery (Li et al., 2016).

## 4 Limitations of ICP-based osmotherapy

In the latest Brain Trauma Foundation, although ICP monitoring is the standard of neurocritical care, intraoperative use is of a rare occurrence. Even if accessible, ICP may not be an accurate assessment of intraoperative brain relaxation, especially in local oedema, venous congestion, or changed compliance. This could be because the measurement of ICP will be significantly altered after the dural opening. The diminished ICP values may reflect a false sense of security. But the potential harm associated with a brain bulge would still be ongoing. Treating numbers instead of operative states, then, risks overtreatment and undertreatment. The indiscriminate use of mannitol also carries well-documented risks: renal impairment and electrolyte imbalance, rebound cerebral oedema, hypotension and hypovolaemia, and increased postoperative complications, reframing brain relaxation as a therapeutic goal (Seo et al., 2017). We suggest intraoperative brain relaxation be embraced as an objective, measurable goal to direct osmotherapy, with three benefits.

• The first advantage is standardisation, as a formal application of established relaxation scales (Pérez de et al., 2022). This scale provides reproducible norms for the use of agents such as mannitol or hypertonic saline.
• The second benefit is reduced subjective variability. Using graded relaxation eliminates the influence of the surgeons' individual preferences or experience.
• The third benefit results in uniform clinical decisions and a focus on patient-centered safety. In other words, reducing unnecessary mannitol decreases exposure to side effects, enhances hemodynamic stability, and enables more patient-centered, personalised anaesthetic management.

## 5 Towards a relaxation-oriented intraoperative protocol

We would prefer a Brain Relaxation–Oriented Strategy (BROS) that includes aspects like the visual grading of relaxation immediately after durotomy, optional subdural pressure monitoring or ultrasound measurement of brain shift, decision to administer osmotherapy only at Grades 3–4 based on thresholds, and a feedback loop considering brain relaxation response and hemodynamic effects. This approach would align intraoperative decision-making with real conditions faced by the surgeon and reduce unnecessary pharmacological interventions.

In conclusion, a paradigm shift towards the management of intraoperative brain relaxation instead of ICP management provides a chance for better management of osmotherapy procedures in neurosurgery. Integration of these procedures with evaluation techniques may result in enhanced outcomes of these surgeries. This paradigm will perhaps have to be authenticated in research to come, together with determination of the influence on operative intervals.

## Disclosures

The authors have no personal, financial, or institutional interest in any of the drugs, materials, or devices described in this article.

## Funding

This study did not receive any funding or financial support.

## Declaration of competing interest

The authors declare that they have no known competing financial interests or personal relationships that could have appeared to influence the work reported in this paper.
